# Editorial—Role of DNA Methyltransferases in the Epigenome

**DOI:** 10.3390/genes10080574

**Published:** 2019-07-30

**Authors:** Albert Jeltsch, Humaira Gowher

**Affiliations:** 1 Department of Biochemistry, Institute of Biochemistry and Technical Biochemistry, University of Stuttgart, 70569 Stuttgart, Germany; 2 Department of Biochemistry, Purdue University, West Lafayette, IN 47907, USA

**Keywords:** DNA methyltransferase function, DNA methyltransferase mechanism, DNA methyltransferase regulation, DNA methyltransferase structure, DNMT1, DNMT3A, DNMT3B, DNA Methylation

## Abstract

DNA methylation, a modification found in most species, regulates chromatin functions in conjunction with other epigenome modifications, such as histone post-translational modifications and non-coding RNAs. In mammals, DNA methylation has essential roles in development by orchestrating the generation and maintenance of the phenotypic diversity of human cell types. This Special Issue of Genes contains eight review articles, which cover several aspects of epigenome regulation by DNA methyltransferases (DNMTs), the enzymes responsible for the introduction of DNA methylation. The manuscripts present the most recent advances regarding the structure and function of DNMTs, their targeting and regulation by interacting factors and chromatin modifications, and the roles of DNMTs in mammalian development and human diseases. However, many aspects of these important enzymes are still insufficiently understood. Potential directions of future work are the regulation of DNMTs by post-translational modifications and their connection to cellular signaling and second messenger cascades on one hand and to large multifactorial epigenetic chromatin circuits on the other. Additionally, technical advancements, including the availability of designer nucleosomes and the rapid development of cryo-electron microscopy are expected to trigger breakthrough discoveries in this exciting field.

## 1. Introduction

DNA methylation at the cytosine—C5 position is found in many species. The methylation is placed in the major groove of double-stranded B-DNA, where it does not interfere with the Watson/Crick base pairing, but it can influence the binding of proteins to specific DNA sequences and thereby, for example, direct the binding of transcription factors to gene regulatory elements. By this mechanism, the methylation adds extra information to the DNA that is not encoded in the DNA sequence and represents one important component of the epigenome [1]. DNA methylation regulates several chromatin functions in conjunction with other epigenome modifications, such as histone post-translational modifications and non-coding RNAs [2]. By orchestrating the generation and maintenance of the phenotypic diversity of the various cell types of the body, DNA methylation plays an essential role in mammalian development [3]. Moreover, DNA methylation provides the substrate for more recently discovered Ten-eleven Translocation (TET) enzymes, which oxidize 5-methylcytosine to the hydroxyl, formyl, and carboxyl state [4]. Numerous studies have demonstrated that aberrant DNA methylation has serious consequences, including the onset and progression of cancer [5,6]. DNA methyltransferases, the enzymes that introduce DNA methylation, clearly are one of the key players in molecular epigenetics [7]. Despite being studied for more than 40 years [8,9,10], recent work has brought important advances in our understanding of the mechanism, function, and regulation of DNA methyltransferases some of which are collected and reviewed in eight publications in this special issue of Genes.

## 2. Structure and Function of DNMTs

Ren et al. [11] describe the newest discoveries regarding the structural basis of DNA methyltransferase 1 (DNMT1) and DNMT3A mediated DNA methylation. Based on recent structure-function investigations of the individual domains or large fragments of DNMT1 and DNMT3A, they review the molecular basis for their substrate recognition and specificity, intramolecular domain–domain interactions, as well as their crosstalk with other epigenetic mechanisms. Their paper highlights the multifaceted nature of the regulation of both DNMT1 and DNMT3A/3B, which is essential for the precise establishment and maintenance of lineage-specific DNA methylation patterns.

## 3. Chromatin Recruitment and Regulation of DNMTs

Jeltsch et al. [12] describe the genomic distribution and variability of DNA methylation in different genomic elements in human and mouse DNA and the connection of DNA methylation with several key histone post-translational modifications, including methylation of H3K4, H3K9, H3K27, and H3K36, and also with nucleosome remodeling. Based on this, they review the mechanistic features of mammalian DNA methyltransferases and their associated factors that recruit these enzymes to genomic sites and mediate the crosstalk between DNA methylation and chromatin modifications.

Laisne et al. [13] describe our current understanding of the recruitment mechanisms of DNA methyltransferase to target sites in mammals. This includes mechanisms of DNMT recruitment by transcription factors, other interacting chromatin modifiers and by RNA. These mechanisms are presented in the context of biologically relevant epigenetic events illustrating how the specific recruitment of DNMTs controls epigenetic signaling.

Xie and Qian [14] and Bronner et al. [15] focus on the specific question of the complex role of UHRF1 in the regulation and targeting of DNMT1. UHRF1 has previously been reported to regulate DNMT1 in multiple ways, including control of substrate specificity and activity based on allosteric regulation of DNMT1, as well as histone and DNMT1 ubiquitylation. Moreover, UHRF1 contributes to the proper genome targeting of DNMT1 by several chromatin interactions with hemimethlyated DNA and modified histone tails. The interplay of these complex multidomain proteins is one illustrative example of the complexity of epigenetic regulation cascades.

## 4. Role of DNMT in Development and Disease

Zeng and Chen [16] integrate these views and review the process of DNA methylation reprogramming during mammalian development. They describe the two waves of DNA methylation reprogramming in mammals occurring in the germline and after fertilization and explain their mechanistic underpinnings. By this they provide an overview of these key reprogramming events, focusing on the important players in these processes including DNA methyltransferases (DNMTs) and TET family of 5mC dioxygenases. Gujar et al. [17] review the role of isoforms of human DNMTs in shaping the epigenome mainly focusing on the DNMT3B isoforms which have documented roles in development and cancer progression, and add another layer of complexity to epigenetic regulation in biological systems.

Norvil et al. [18] describe the effect of disease-associated germline mutations in DNMTs. Recent advances in whole genome association studies have helped to identify mutations and genetic alterations of DNMTs in various diseases that have the potential to affect the biological function and activity of these enzymes. Several of these mutations are germline-transmitted and associated with a number of hereditary disorders, including neurological dysfunction, growth defects, and inherited cancers. This review describes DNMT mutations that are associated with rare diseases, the effects of these mutations on enzyme activity and provides insights on their potential effects based on the known crystal structure of these proteins.

## 5. Conclusions and Outlook

While this collection of review articles illustrates in an impressive manner the high level of mechanistic understanding of DNMTs that has been reached over the last decade of research, it also emphasizes the gaps and open questions in the field that need to be answered. It is anticipated that, in future, the investigation of the targeting and regulation of DNMTs will be intensified to finally understand the mechanisms leading to the generation of DNA methylation patterns during early development, germ cell development and onset of disease. In this respect, the details of the regulation of DNMTs by post-translational modifications are still uncovered, as well as their connection to cellular signaling and second messenger cascades. The increasing availability of designer nucleosomes will allow powerful enzymatic in vitro studies regarding the recruitment and crosstalk of DNMTs with other chromatin marks. The further improvement of cryo-electron microscopy will enable structural investigation of larger protein complexes with atomic resolution, allowing to study the structure and conformation of DNMTs also in complex with their regulatory factors.
